# Dogs wait longer for better rewards than wolves in a delay of gratification task: but why?

**DOI:** 10.1007/s10071-020-01346-7

**Published:** 2020-02-14

**Authors:** Friederike Range, Désirée Brucks, Zsófia Virányi

**Affiliations:** 1 Wolf Science Center, Domestication Lab, Konrad Lorenz Institute of Ethology, University of Veterinary Medicine, Savoyenstraße 1a, 1160 Vienna, Austria; 2 Comparative Cognition, Messerli Research Institute, University of Veterinary Medicine, Medical University of Vienna, University of Vienna, Vienna, Austria; 3 Institute for Agricultural Sciences, ETH Zurich, Zurich, Switzerland

**Keywords:** Behavioural strategies, Domestication, Intertemporal choice, Quality exchange paradigm, Self-control

## Abstract

Self-control has been shown to be linked with being cooperative and successful in humans and with the *g*-factor in chimpanzees. As such, it is likely to play an important role in all forms of problem-solving. Self-control, however, does not just vary across individuals but seems also to be dependent on the ecological niche of the respective species. With dogs having been selected to live in the human environment, several domestication hypotheses have predicted that dogs are better at self-control and thus more tolerant of longer delays than wolves. Here we set out to test this prediction by comparing dogs’ and wolves’ self-control abilities using a delay of gratification task where the animals had to wait for a predefined delay duration to exchange a low-quality reward for a high-quality reward. We found that in our task, dogs outperformed the wolves waiting an average of 66 s vs. 24 s in the wolves. Food quality did not influence how long the animals waited for the better reward. However, dogs performed overall better in motivation trials than the wolves, although the dogs’ performance in those trials was dependent on the duration of the delays in the test trials, whereas this was not the case for the wolves. Overall, the data suggest that selection by humans for traits influencing self-control rather than ecological factors might drive self-control abilities in wolves and dogs. However, several other factors might contribute or explain the observed differences including the presence of the humans, which might have inhibited the dogs more than the wolves, lower motivation of the wolves compared to the dogs to participate in the task and/or wolves having a better understanding of the task contingencies. These possible explanations need further exploration.

**Electronic supplementary material:**

The online version of this article (10.1007/s10071-020-01346-7) contains supplementary material, which is available to authorized users.

## Introduction

The ability to stop an immediate behaviour in favor of a more advantageous behaviour has been termed ‘inhibitory control’. Being able to withhold such actions is highly important for all kinds of social interactions and decision-making processes. However, previous studies have demonstrated that there are different aspects of inhibition (e.g. Bray et al. 2014; Brucks et al. 2017a, b). One of them is self-control, defined as the capacity to reject an immediate reward in favour of a delayed but better reward. Self-control or delay of gratification abilities have received a lot of attention, because, in humans, better self-control is linked to being healthier, wealthier, and less involved in criminal actions (Moffitt et al. 2011), more cooperative (Giannotta et al. 2011) and better at problem-solving (Diamond 1990). Furthermore, a recent study in chimpanzees revealed a link between self-control and the general intelligence—‘*g*’—factor (Beran and Hopkins 2018), highlighting the possibly universal importance of this ability.

Self-control is usually measured by applying an exchange paradigm in which individuals need to resist taking an initial low-value reward for a certain amount of time before they are offered the chance of exchanging it for a better reward. Performance in delay of gratification tasks seems to be affected by several factors. For example, animals seem to be better in tolerating higher delays when the delayed reward is of better quality rather than of higher quantity (e.g. Wascher et al. 2012; Auersperg et al. 2013; Hillemann et al. 2014; Brucks et al. 2017a, b). Moreover, individuals vary in their performance (Wascher et al. 2012; Auersperg et al. 2013; Hillemann et al. 2014; Brucks et al. 2017b), which seems to be at least partly dependent on the behavioural patterns adopted during the delay (Evans and Beran 2007; Koepke et al. 2015). For example, human children that were able to distract themselves by playing outperformed children that focused on the reward (Steelandt et al. 2012). Similarly, dogs that dozed or looked away performed better than dogs that gazed at the rewards (Brucks et al. 2017b).

However, differences in delay of gratification abilities occur not only across individuals but also across species (e.g. Amici et al. 2008; Lakshminaryanan and Santos 2009; Stevens et al. 2005), suggesting that specific selection pressures of the environment of the respective species contribute to this variation. An interesting model system to investigate this variation are wolves and dogs, since, while being closely related, they differ in regard to their ecological niche. Dogs are a domesticated species and for thousands of years they have been selected for a life around humans (Coppinger and Coppinger 2016) as well as for specific traits to cooperate with us. More specifically, it has been suggested that during the process of domestication, dogs were selected against fear and aggression, and consequently, dogs possess a less reactive and tamer temperament than their undomesticated relatives, wolves (‘Emotional reactivity hypothesis’, Hare and Tomasello 2005). Selection on systems mediating aggression might increase inhibitory control abilities as well, as demonstrated by more aggressive rats being worse at tolerating delayed rewards than less aggressive rats (Van den Bergh et al. 2006). Similarly, impulsive dogs often develop behavioural problems, such as impulsive aggression (e.g. Fatjó et al. 2005), which can additionally be triggered by fear (Archer 1976). Accordingly, animals that can inhibit their fearful and/or aggressive responses might have had an advantage when interacting with humans (see Gácsi et al. 2005; Gácsi et al. 2009a, b). Indeed ‘the synergistic hypothesis’ of domestication explicitly proposes that compared to wolves, dogs may show superior abilities in certain communicative tasks with humans (e.g. the pointing task) due to the fact that they are more inclined to inhibit their immediate reactions in favour of delayed rewards (Gácsi et al. 2009a, b). Accordingly, these domestication hypotheses posit that dogs shall outperform wolves in inhibition tasks as well as in a delay of gratification task.

To date, few studies have set out to specifically compare inhibitory control abilities between wolves and dogs. One study compared human-socialized wolves’ and dogs’ inhibitory control abilities in two different detour tasks (Marshall-Pescini et al. 2015). Interestingly, while the wolves were better than dogs in a ‘detour’ task, where they had to walk around a V-shaped fence to reach the food reward in the middle, the dogs outperformed the wolves in the ‘cylinder’ task. This latter task involved first a few training trials, where the animals learnt to stick their head into either end of an opaque cylinder to get access to a food reward. After the training trials, the animals were then confronted with a transparent tube and had to avoid touching the exterior of the cylinder with any part of their head or paw before sticking their snout in at either side despite seeing the food. Another study conducted a battery of motor and cognitive inhibition tasks (Brucks et al. 2019). Like in other studies (Bray et al. 2014; Müller et al. 2016; Brucks et al. 2017a, b), the single inhibition tests did not correlate with each other either in dogs or wolves, suggesting that inhibitory control is context-specific in both canine groups. Moreover, dogs and wolves differed in neither of the three components (motivation, flexibility and perseveration) that were found to explain the variation of behaviours across tests (Brucks et al. 2019). In contrast, a study testing wolves and dogs in a pointing task (Gácsi et al. 2005; Gácsi et al. 2009a, b) found that dogs struggled less than wolves when being held by a human and thus showed better inhibitory control abilities than wolves when being socially constrained.

No study has yet compared wolves’ and dogs’ performance in a delay of gratification task, even if pet dogs have been tested in two prior studies. In the first study, which required the dogs to go through a lengthy training process, the dogs were surprisingly good, tolerating delays of up to 18 min, thus outperforming many primate species (Leonardi et al. 2012). In the second study, where training was kept to a minimum and the experimenter and owner were hidden behind a curtain during the actual test, dogs showed a much lower tolerance for delays at a group-level. Only half of the dogs could wait for more than 10 s in a quality condition and 2 s in a quantity condition. However, some dogs were exceptional and waited up to 140 s for a better reward in both conditions, with one dog succeeding with a delay of 15 min (Brucks et al. 2017b).

Here we tested human-socialized wolves and dogs that have had the same experience across life in a quality exchange paradigm in which individuals were required not to eat an available low-value reward for the waiting duration before they were offered the chance to return the low-value reward for a reward of higher value. To better understand possible differences in the performance of wolves and dogs, we investigated the influence of the reward type (sausage vs. meat), the behavioural strategies and their motivation to participate in the task. Following the domestication hypotheses, we predicted that dogs would tolerate higher delays compared to wolves. We expected that animals would be more inclined to wait longer for more preferred food and we expected meat to be overall preferred. Furthermore, we expected that more successful animals would use alternative strategies like looking away from the food to distract themselves. While it is possible that both species perform equally well with low delay times, the differences should become detectable in higher delay durations.

## Methods

## Ethical statement

No special permission for use of animals (wolves) in such sociocognitive studies is required in Austria (Tierversuchsgesetz 2012–TVG 2012). The relevant committee that allows running research without special permissions regarding animals is: Tierversuchskommission am Bundesministerium für Wissenschaft und Forschung (Austria).

### Subjects

We tested eight timber wolves and five mixed-breed dogs in this study (see Table 1 for details). The animals were similarly raised and kept at the Wolf Science Centre in Ernstbrunn, Austria. All tests were carried out between March 2010 and April 2017 in an indoor test room. The tests were conducted by two trainers, who were familiar to the animals and interacted with them on a daily basis. Raw data are available as supplementary data.

**Table 1:** Individual characteristics of wolves and dogs that participated in the study

Name	Species	Sex	Age (years)
Amarok	Wolf	M	1.8
Aragorn	Wolf	M	5.7
Chitto	Wolf	M	1.8
Geronimo	Wolf	M	0.8
Kaspar	Wolf	M	5.6
Nanuk	Wolf	M	0.9
Shima	Wolf	F	6.3
Tala	Wolf	F	1.9
Asali	Dog	M	1.1
Bashira	Dog	F	1.1
Binti	Dog	F	1.1
Hakima	Dog	M	1.1
Meru	Dog	M	1.1

### Apparatus and experimental setup

**Fig. 1:**
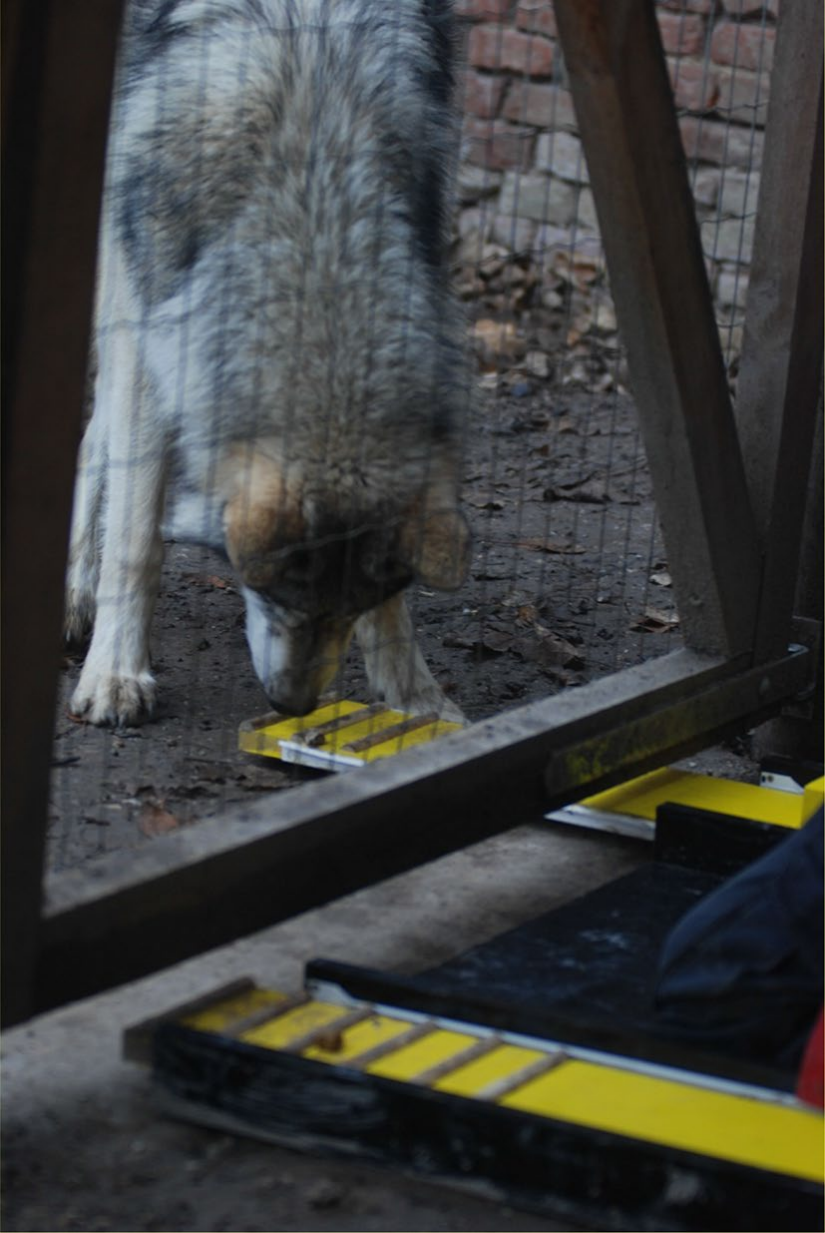
Test set-up. Picture of a wolf working on the apparatus

A wooden frame covered with metallic wire was installed in a corner of the indoor test room. The frame was positioned 10 cm from the ground, hence leaving space for the test apparatus under the fence while preventing the animals from gaining access to the food rewards behind the fence. The animals could freely move in the testing room. The test apparatus consisted of two wooden panels that ran on rails, which were fixed to a wooden board (Fig. 1).

One of the experimenters sat behind the fence, kneeling on the wooden board. She managed the reward distribution and manipulated the panels during testing. The second experimenter filmed from a corner of the test room. Based on the wolves’ and dogs’ food preferences in the food preference tests (see below), dry food was used as low-value rewards and sausage and meat were used as high-value rewards.

### Training

The animals were first trained to push back the panels with their nose using a shaping and positive reinforcement method. After they learned to push back the panels, they were trained to exchange a stone for a piece of dry food increasing the delay to 2 s using the standard experimental procedure adopted in delay of gratification tests (Anderson et al. 2010; Vick et al. 2010; Dufour et al. 2012; Leonardi et al. 2012; Koepke et al. 2015; Brucks et al. 2017b). Once they successfully exchanged the stone for the dry food, they were trained to exchange the dry food for a piece of meat. When an individual succeeded to wait for the higher quality reward (HQR) in 7 of the 8 trials within a session, it proceeded to the test phase (see Table 2 for summary of procedures).

**Table 2:** Overview test procedures

Phase	Sessions	Trials	Food type	Delay (sec)	Criterion
Food preference test	Beginning of each test session	4	Dry food vs. Sausage	0	No
		4	Dry food vs. Meat		
Delay of gratification test	Six sessions per delay stage^a^	Motivational trials			
		2	Dry food vs. Sausage	2	No
		2	Dry food vs. Meat		
		Delay trials			
		2	Dry food vs. Sausage	2, 5, 10, 15, 20, 25, 35, 45, 60	Wait in ≥ 1 delay trial
		2	Dry food vs. Meat		

^a^Except for last delay stage, if individual did not succeed in a single trial within two sessions

### Test

#### Food preference test

At the beginning of each test session, the animals were presented with eight trials having a choice of sausage or meat, cut into equally sized pieces of approximately 1 cm. The experimenter sat behind the fence and presented the food types to the subject by lifting the food hidden in her hands (one food type per hand). After the animal had sniffed both food types, the food was placed on plates on each side of the experimenter. One reward was placed on the plate first, while the other reward remained in the hand of the experimenter, and after a short interval of 2 s was placed on the other plate. The order of the reward, which was put down first was randomized and counterbalanced across trials. Both plates were pushed in front of the fence simultaneously and the animal was free to eat one piece of food (see Video for procedure). A choice was noted when the animal approached and ate from one plate. As soon as the animal chose one plate, the experimenter pulled the other plate back behind the fence. Eight trials with randomly changing sides of the rewards were conducted.

#### Delay of gratification test procedure

After the food preference test, the delay of gratification test was started. The experimenter was kneeling behind the fence in-between the panels and held up both reward types (LQR and HQR) in her hands. The sides of the rewards were semi-randomized (i.e. not more than twice in a row on the same side) and counterbalanced within test sessions. The rewards were held at the height of the animal’s head close to the fence so that the subject could sniff them for 2 s. Then the LQR was put on a panel and the panel was pushed out within reach of the subject. The HQR remained in the experimenter’s hand and was held up for the entire delay duration (see Video for procedure). The reward in the right hand was always placed on the right panel and the reward in the left hand on the left panel. Eye contact with the animals was avoided and the experimenter looked to the floor throughout testing. If the animal did not consume the LQR during the delay duration, the HQR was placed on the second panel. The animal was then allowed to push back the first panel (with the LQR on it), and in return, the HQR on the second panel was pushed into reach of the animal. However, if the animal consumed the LQR, the HQR was immediately lowered and put back into the food container before the next trial started (inter-trial interval: 10 s; see Video of procedures). In case the animal tried to push back the low-value reward during the waiting time, the experimenter rendered the action unsuccessful by holding the panel in place. In case the animals failed to exchange the LQR (i.e. eating LQR instead of pushing the panel back) after the delay duration was completed, it was noted as a failure. Finally, in case the animal did not push the panel back or/and did not eat the LQR, the trial was repeated since it only happened if the animals were distracted.

Within a test session, two different types of trials were conducted: four motivation trials with a fixed delay duration of 2 s, and four delay trials with an increasing delay duration. Motivation trials were included to check whether the animals were sufficiently motivated to participate in the experiment. In the delay trials, the delay between the immediately accessible low-quality reward and the delayed high-quality reward was increased in consecutive steps (2 s, 5 s, 10 s, 15 s, 20 s, 25 s, 35 s, 45 s, 60 s, 75 s, 100 s, 150 s, 175 s) across sessions.

Test sessions were conducted on different days and each test session consisted of eight trials, which were tested in random order. The trials additionally differed in the type of reward that was presented: dry food vs. sausage or dry food vs. meat. Accordingly, each trial type (motivation/delay) was tested twice with sausage, and twice with meat as the HQR. Six test sessions were conducted per delay stage (except the very last one). If the subject was successful (i.e. waiting for HQR) in at least one trial within the six sessions, it was tested in the next step with a higher delay. However, if the animal passed to the next delay stage having succeeded only in one trial across the six sessions, we stopped after two sessions in the next delay stage, if the animal was not once successful at all to avoid frustration in the animals. All test sessions were videotaped. An example of a test session is included as a supplementary video.

### Analyses

We coded the following variables: choice of reward (frequency: low (LQR) or high (HQR), posture (duration: stand, sit, lay); proximity to rewards [duration: close (within 20 cm), medium (20–80 cm), distant (more than 80 cm)], attention [duration: look away (turning head away from fence), gaze alternations (frequency: alternations between looking at low and high reward)], and locomotion (duration: movement with at least two paws leaving the ground). Moreover, if an animal waited for the delayed option but failed to exchange the LQR, this was coded as a failure to exchange (this happened in 0.10 ± 0.36 of all trials). Unfortunately, 9.5% of the videos were missing. For those sessions without video records, we could only use the success rate for the analyses since that was coded during the live sessions, but could not include the behaviours.

The data was analysed in R (R Core Team 2014) using the packages ‘lme4′ (Bates et al. 2014) and ‘nlme’ (Pinheiro et al. 2008). We assessed individual food preferences by running binomial tests to see whether the choice for either option deviates from chance level (0.5). Furthermore, linear models (LM) were run to reveal if the animals developed preferences throughout test sessions.

Secondly, a generalized-least square model (GLS) was run to investigate whether the reward type affected the waiting performance (Model 1). Success rate (i.e. number of trials waiting for delayed reward/total number of trials) was used as response variable, to control for seven sessions in which only three instead of four delay trials were conducted. This model included the fixed effects reward type (factor: meat, sausage), delay, species and interactions between these variables, with identity of the animals as random effect.

Furthermore, linear mixed models (LMMs) were utilized to analyse the influence of delay (factor: 2, 5, 10, 15, 20, 25, 35, 45, 60 s), of the number of test sessions within a certain delay period, and of the behavioural variables on the success rate in the delay trials (Model 2). For investigating whether dogs and wolves differed in their success, this model included an interaction between species and delay. To enter the behavioural variables into the model, we calculated their proportions (i.e. frequency or duration/total duration of test session), as the test sessions had different durations (due to the increasing delay durations). The model included proportions of gaze alternations, looking away and locomotion as fixed factors.

Finally, to see if the animals were motivated to participate in the task, a generalized least square model (GLS) was used to analyse the success rate in the motivational trials (i.e. waiting for delayed option/total number of motivational trials) due to its non-normal distribution (Model 3). This model included species and delay in the delay trials of the same session as fixed effects.

All models were selected based on stepwise backward regression analyses using likelihood ratio tests (LRT) to retain only significant effects in the final models.

Three coders coded the videos, with two coding 27% each, while one main coder coded 46% of the videos. To get a measure of reliability between coders, the main coder coded additionally 20% of the videos of the two other coders (ICC: latencies: coder 1 < 0.99, coder 2 < 0.87; look away: coder 1 = 0.80, coder 2 = 0.79; locomotion: coder 1 = 0.97, coder 2 = 0.89; Cohen’s kappa: gaze alternations: coder 1 = 0.82, coder 2 = 0.89).

## Results

### Food preferences

When combining the individual choices between sausage and meat across all test sessions, we found that two wolves (Chitto, Geronimo) and one dog (Meru) exhibited a preference for meat (binomial tests: *p* < 0.035), while the other individuals chose randomly (binomial tests: *p* > 0.144). Moreover, some individuals developed a preference for either of the two options across test sessions. In particular, two wolves chose meat more often with increasing session numbers (LM: Tala: 0.11 ± 0.03, *t* = 3.11, *p* = 0.005; Chitto: 0.07 ± 0.03, t = 2.24, *p* = 0.034), while two wolves and one dog increased their sausage choices across test sessions (LM: Kaspar: − 0.06 ± 0.02, *t* = − 3.67, *p* < 0.001; Aragorn: − 0.05 ± 0.02, *t* = − 3.28, *p* = 0.002; Hakima: 0.06 ± 0.01, *t* = − 6.38, *p* < 0.001). All other individuals did not show any preferences between sausage and meat and their choices did not change across sessions.

### Species

**Fig. 2:**
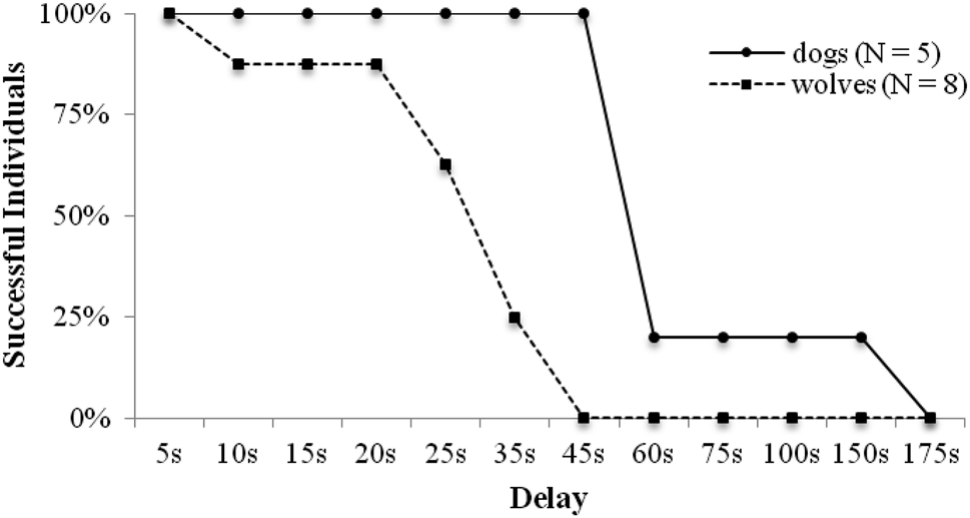
Performance of individuals in the different delays. The figure shows the percentage of successful individuals across delay stages plotted separately for dogs and wolves

Dogs tolerated a maximum delay of 45 s (mean: 66.00 ± 46.96 s), whereas the wolves tolerated only a maximum delay of 20 s (mean: 23.75 ± 9.54 s, Fig. 2). The model revealed a species *x* delay interaction (Model 1; *χ*^2^ (6) = 109.1, *p* < 0.001), which indicated that dogs were more successful than wolves in all delay stages (see Table 3).

**Table 3:** Effects of delay duration and species on success in delay trials (Model 2)

Fixed effect	Estimate	S.E.	*df*	*t* value	*p*
Intercept	2.57	0.42	12.1	6.08	< 0.001**
Delay 10 s	0.03	0.19	442.2	0.18	0.854
Delay 15 s	− 0.11	0.19	442.2	− 0.55	0.582
Delay 20 s	0.35	0.19	442.2	1.84	0.067
Delay 25 s	0.21	0.19	442.2	1.10	0.271
Delay 35 s	− 0.63	0.19	442.2	− 3.31	0.001**
Delay 45 s	− 1.17	0.19	442.2	− 6.15	< 0.001**
Delay 60 s	− 1.52	0.24	442.4	− 6.35	< 0.001**
Delay 75 s	− 0.57	0.34	443.3	− 1.67	0.096
Delay 100 s	− 0.22	0.34	443.3	− 0.65	0.516
Delay 150 s	− 2.84	0.34	443.3	− 8.28	< 0.001**
Delay 175 s	− 3.36	0.55	442.6	− 6.16	< 0.001**
Species: Wolf	− 0.24	0.54	12.1	− 0.45	0.661
Delay 10 s × Wolf	− 0.66	0.25	444.4	− 2.68	0.008**
Delay 15 s × Wolf	− 0.58	0.25	444.4	− 2.31	0.021*
Delay 20 s × Wolf	− 1.25	0.25	444.4	− 5.02	< 0.001**
Delay 25 s × Wolf	− 1.89	0.26	444.5	− 7.42	< 0.001**
Delay 35 s × Wolf	− 2.37	0.28	444.4	− 8.33	< 0.001**
Delay 45 s × Wolf	− 2.08	0.44	443.6	− 4.67	< 0.001**

* *p* < 0.05

** *p* < 0.01

### Factors influencing success

#### Reward types

The type of delayed reward (meat or sausage) did not affect the waiting success (Model 1; type *x* delay: Anova: *F*_12,964_ = 0.25, *p* = 0.995), neither in dogs nor in wolves (Anova: type *x* species: Anova: *F*_1964_ = 0.34, *p* = 0.563).

#### Behavioural patterns

Dogs and wolves spent most of their time standing in close proximity to the rewards (proportion close: mean ± SE: 0.97 ± 0.09; medium: 0.01 ± 0.06; distant: 0.01 ± 0.06; and proportion stand: 0.98 ± 0.07; sit: 0.01 ± 0.06; lay: 0.00 ± 0.01); due to this low variation, we were not able to include body posture and proximity to food into the analyses. Since we found a significantly better performance of dogs in all delay stages (see Table 3), we analysed what influences the ability to delay gratification separately for dogs and wolves (Model 2).

#### Dogs (Model 2.1)

The dogs’ success did not change across test sessions using the same delay (see Table 4), but started decreasing at the delay stage of 35 s (see Table 5). Moreover, the number of gaze alternations between the immediately available reward and the delayed reward, as well as looking away from the rewards did not affect their waiting performance (see Table 4). However, we found an interaction between delay and locomotion (see Table 4), which seemed to affect dogs’ waiting performance (see Table 5). The less the dogs moved during the trial, the more successful they were in waiting for the delayed option at least during the lower delay stages (up to 45 s). The opposite effect was observed during the higher delay stages (from 60 s on), in which increased locomotion facilitated waiting success. However, this effect was driven by the one dog, which reached the 175 s delay stage, while all other dogs dropped out at 60 s. Interestingly, the dogs moved significantly less than the wolves during the waiting period (Wilcoxon test: *T* = 41,700, *N* = 422, *p* < 0.001).

**Table 4:** Summary of effects of delay, locomotion, looking away, gaze alternations between low-quality food and high-quality food, as well as test session on dogs’ and wolves’ success in the exchange task

Dogs		Wolves	
Delay *x* locomotion	*χ*^2^ (10) = 36.90, *p* < 0.001**	Delay *x* gaze alternations	*χ*^2^ (6) = 20.46, *p* = 0.002**
Look away	*χ*^2^ (1) = 1.08, *p* = 0.300	Look away	*χ*^2^ (1) = 8.98, *p* = 0.003**
Gaze alternations	*χ*^2^ (1) = 0.25, *p* = 0.117	Locomotion	*χ*^2^ (1) = 0.03, *p* = 0.864
Session	*χ*^2^ (1) = 0.19, *p* = 0.661	Session	*χ*^2^ (1) = 6.93, *p* = 0.011*

Results from Wald *χ*^2^ tests are depicted (Model 2.1 for dogs and model 2.2 for wolves)

* *p* < 0.05

** *p* < 0.01

**Table 5:** Effects of delay, and locomotion on dogs’ success in delay trials (Model 2.1; Linear mixed model output after model selection)

Fixed effect	Estimate	S.E.	*df*	*t* value	*p*
Intercept	0.94	0.14	76.4	6.57	< 0.001**
Delay 10 s	0.04	0.15	200.4	0.28	0.777
Delay 15 s	0.04	0.15	200.4	0.31	0.761
Delay 20 s	0.01	0.15	201.3	0.05	0.962
Delay 25 s	− 0.07	0.15	201.2	− 0.46	0.649
Delay 35 s	− 0.15	0.15	201.2	− 1.03	0.304
Delay 45 s	− 0.36	0.15	201.2	− 2.46	0.015*
Delay 60 s	− 0.74	0.16	200.9	− 4.63	< 0.001**
Delay 75 s	− 0.27	0.27	200.9	− 0.97	0.334
Delay 100 s	− 0.10	0.25	200.7	− 0.41	0.679
Delay 150 s	− 1.28	0.31	200.7	− 4.14	< 0.001**
Delay 175 s	− 1.15	0.61	200.2	1.87	0.063
Locomotion	− 0.21	0.28	201.6	− 0.77	0.445
Delay 10 s × locomotion	− 0.38	0.36	200.3	− 1.06	0.289
Delay 15 s × locomotion	− 1.46	0.54	200.5	− 2.71	0.007**
Delay 20 s × locomotion	0.11	0.69	201.1	0.15	0.879
Delay 25 s × locomotion	0.51	0.53	201.2	0.99	0.323
Delay 35 s × locomotion	− 2.33	0.67	200.7	− 3.47	< 0.001**
Delay 45 s × locomotion	− 0.88	0.55	201.1	− 1.59	0.115
Delay 60 s × locomotion	0.98	0.46	200.4	2.13	0.035*
Delay 75 s × locomotion	0.61	2.54	200.0	0.24	0.812
Delay 100 s × locomotion	NA	NA	NA	NA	NA
Delay 150 s × locomotion	2.75	2.24	200.0	1.23	0.222
Delay 175 s × locomotion	0.21	6.30	200.0	0.03	0.973

* *p* < 0.05

** *p* < 0.01

#### Wolves (Model 2.2)

The general success decreased with increasing delay duration and also with increasing session numbers within the same delay stage (see Table 4). We found an interaction between gaze alternations and delay stage (see Table 4). Gaze alternations between the low quality reward and the high quality reward was associated with increasing waiting success, in particular in the lower delay stages (see Table 6). Moreover, looking away from the rewards was associated with higher waiting success in the wolves (see Table 6). In comparison to the dogs, the wolves looked away from the rewards less often (Wilcoxon test: *T* = 12,528.5, *N* = 405, *p* = 0.001) and exhibited more gaze alternations between the reward options (Wilcoxon test: *T* = 20,350.5, *N* = 405, *p* < 0.001). The wolves’ waiting success was not affected by locomotion (see Table 4).

**Table 6:** Effects of delay, gaze alternations, looking away and session number on wolves’ success in delay trials (Model 2.2; Linear mixed model output after model selection)

Fixed effect	Estimate	S.E.	*df*	*t *value	*p*
Intercept	0.81	0.15	11.48	5.57	< 0.001**
Delay 10 s	− 0.28	0.08	183.6	− 3.38	< 0.001**
Delay 15 s	− 0.42	0.09	184.0	− 4.76	< 0.001**
Delay 20 s	− 0.28	0.09	183.7	− 3.19	< 0.001**
Delay 25 s	− 0.58	0.10	183.7	− 6.16	< 0.001**
Delay 35 s	− 1.11	0.12	181.9	− 9.03	< 0.001**
Delay 45 s	− 1.09	0.31	180.3	− 3.47	< 0.001**
Gaze alternations	0.06	0.16	183.7	0.40	0.240
Look away	0.68	0.23	182.3	2.97	0.003**
Session	− 0.02	0.01	180.0	− 2.56	0.011*
Delay 10 s × alternations	0.26	0.22	181.1	1.18	0.240
Delay 15 s × alternations	0.99	0.30	181.4	3.32	0.001**
Delay 20 s × alternations	− 0.43	0.30	181.1	− 1.46	0.146
Delay 25 s × alternations	− 0.13	0.32	180.9	− 0.43	0.666
Delay 35 s × alternations	0.46	0.49	180.3	0.93	0.353
Delay 45 s × alternations	− 1.08	5.19	179.9	− 0.21	0.835

* *p* < 0.05

** *p* < 0.01

#### Motivational trials (model 3)

**Fig. 3:**
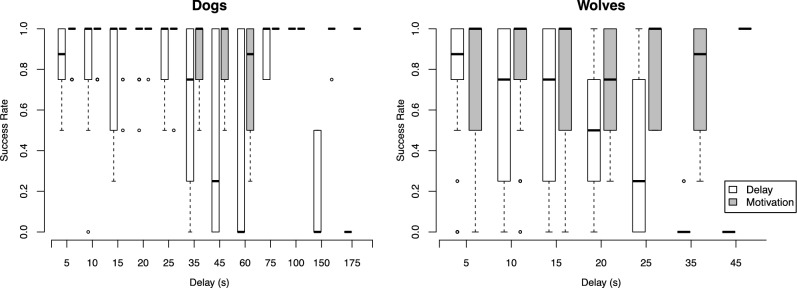
Success rates of dogs and wolves. Success rate in delay (white) and motivational trials (grey) across delay stages in addition to Tables 3 and 4

Dogs and wolves differed in their success in the motivational trials (Anova: *F*_1471_ = 58.91, *p* < 0.001), with wolves being less successful than dogs (GLS: − 0.16 ± 0.02, *t* = − 7.67, *p* < 0.001; see Fig. 3). In dogs, the performance in the motivational trials was affected by the delay duration of the delay trials in the same session (Anova: *F*_11,231_ = 3.79, *p* < 0.001). In particular, dogs stopped waiting in the motivational trials when the delay duration of the delay trials increased (and their success in the delay trials decreased; see Table 7; Fig. 3) suggesting that they did not differentiate between these trials. Interestingly, the dog that reached the highest delay stage did not stop waiting in the motivational trials with decreasing success. No such effect of the delay duration on the waiting success in the motivational trials was observed among the wolves (Anova: *F*_6222_ = 0.76, *p* = 0.605) and wolves continued to succeed in these trials independent of the performance in the delay trials (see Fig. 3).

**Table 7:** Effects of delay duration on success in motivational trials in dogs (Model 3; output generalized-least-squares model after model selection)

Fixed effect	Estimate	S.E.	*t* value	*p*
Intercept	0.96	0.02	42.78	< 0.001**
Delay 10 s	0.00	0.03	0.00	1.000
Delay 15 s	0.01	0.03	0.26	0.795
Delay 20 s	0.03	0.03	1.04	0.299
Delay 25 s	0.03	0.03	0.78	0.436
Delay 35 s	− 0.04	0.03	− 1.30	0.195
Delay 45 s	− 0.07	0.03	− 2.08	0.039*
Delay 60 s	− 0.17	0.04	− 4.30	< 0.001**
Delay 75 s	0.04	0.06	0.75	0.454
Delay 100 s	0.04	0.06	0.75	0.454
Delay 150 s	0.00	0.06	0.00	1.000
Delay 175 s	0.04	0.09	0.46	0.646

* *p* < 0.05

** *p* < 0.01

## Discussion

In the current study, we found that wolves and dogs differed in their ability to delay gratification in an exchange task administered by a familiar human person. While both adult wolves and adult dogs could delay gratification in a quality condition, dogs outperformed wolves with tolerating a mean delay of 66 s compared to 23 s. There are a few interesting aspects of this study that warrant further discussion.

Comparing our dog results with previously published research on dogs, while one dog in each study tolerated similarly high delays, the mean delay tolerated by our dogs was almost twice as long as the one reached by the 12 pet dogs in Brucks et al. (2017b) study. Interestingly, in the pet dogs, there was a very high variance between individual dogs with 7 of the 12 dogs waiting only up to 20 s. In our smaller sample, variance was rather small with all five dogs waiting at least 45 s (and seven of eight wolves waiting at least 20 s). There are two possible, likely additive explanations for this difference between the two studies. Firstly, our animals at the Wolf Science Center are kept under more standardized conditions (including the training procedures and life experiences) than pet dogs that receive individualized training by their owners and have vastly variable life experiences. Secondly, we tested the animals face to face with one of our animal trainers, while in the study with pet dogs the experimenter and owner were hidden behind a cover and not visible during the test. Thus, our dogs might have perceived our set-up more as an obedience task compared to the pet dog studies, which could, in connection with the standardized training in our facility, lead to higher social inhibition (see Range et al. 2019; Udell 2015), which in this paradigm would seem like better self-control. A similar explanation has also been put forward to explain the long delays tolerated by the pet dogs in the study of Leonardi et al. (2012)*,* in which two of the five participating dogs belonged to the experimenter and were tested face to face*,* in comparison to the pet dogs in Brucks et al. (2017b).

Interestingly, this latter explanation could, at least partly, also explain the difference we observed between wolves and dogs in this paradigm, with dogs clearly tolerating longer delays than wolves. While both wolves and dogs are very attentive and cooperative towards humans, dogs follow the lead of the humans more than wolves, suggesting that dogs might have been selected for increased submissive inclinations (Deferential Behaviour Hypothesis, Range et al. 2019) to minimize conflicts. A potential difference in submissiveness, especially towards humans, could, in our task with the human experimenter possessing the food and directly facing the animal, lead to better social inhibition/self-control in the dogs than the wolves. Interestingly, also in the other studies where the dogs outperformed wolves in terms of inhibitory control, humans interacted with the animals by constraining them (pointing: Gácsi et al. 2009a, b) or repeatedly demonstrating an action (cylinder task: Marshall-Pescini et al. 2015). In contrast, when the animals were tested in more asocial settings (detour task: Marshall-Pescini et al. 2015; inhibitory control battery: Brucks et al. 2019) or where the animal needed to wait for a conspecific partner (Marshall-Pescini et al. 2017), wolves did at least as well as if not better than dogs (see also Ostojić and Clayton 2014). However, when comparing the wolves’ performance with the performance of the pet dogs in the more asocial delay of gratification task (Brucks et al. 2017b), the dogs still outperform the wolves with 5 of the 12 dogs waiting for more than 20 s and as a group, tolerating a mean delay of 35.6 s. Interestingly, the variance of the pet dogs in this self-control task is rather high, and it would be important to know whether the dogs that performed better had some additional training allowing them to tolerate higher delays. Also, while the owner and experimenter were not in visual contact during testing, they were both present in the room and thus could have still influenced the behaviour of the pet dogs, especially if extensively trained.

Thus, two factors might influence the results in our task. On the one hand, self-control abilities of wolves and dogs might indeed vary. On the other hand, social and non-social inhibition might potentially be separate constructs, with domesticated species likely being stronger socially inhibited by humans than non-domesticated species (Brucks et al. 2019). Unfortunately, the current study was run before the more recent ones and the chosen set-up does not allow us to differentiate between these two possibilities. However, independently of the reason, the behaviour of dogs being more self-controlled in the presence of the humans, is likely one reason, why dogs make better pets than wolves.

Another possibility, why wolves performed worse compared to dogs in this task, is their much higher activity level. Several studies have shown that wolves are more explorative and active than dogs (Rao et al. 2018a, b), potentially making it difficult for the animals to inhibit manipulating the apparatus. Also in this task, wolves were more active than the dogs (wolves 50.4 ± 16.8% and dogs 17.3 ± 17.4% of delay time active). The dogs’ inactivity could potentially be explained by them being socially inhibited by the human if indeed dogs see the situation as an obedience task, as suggested above. This would also explain why the dogs’ success increased when being less active. For the wolves, in contrast, there was no effect of locomotion on the wolves’ ability to delay gratification (see Table 5 for summary of results).

Both dogs and wolves used variable, albeit partly different, strategies to distract themselves during the delay periods. Dogs and to a lesser degree wolves did look away from the food, as has been reported also for other species including children (Mischel and Ebbesen 1970), chimpanzees (Evans and Beran 2007), a grey parrot (Koepke et al. 2015) and pet dogs (Brucks et al. 2017b). Moreover, wolves alternated their gaze between the high- and low-value reward, which increased their success (see Table 5 for summary of results). This points to wolves paying very close attention to what is happening when they are actually looking. It has already been proposed that the wolves pay attention to details more than dogs (Range et al. 2014; Range and Virányi 2014). Interestingly, though the animals would have been able to distract themselves in our set-up by approaching and trying to interact with the familiar person who was holding the camera, neither dogs nor wolves used this option even once but completely ignored that person. This is interesting since chimpanzees have been reported to engage in self-distracting behaviours and interact with toys when giving the option to increase their waiting times (Evans and Beran 2007). It is possible that our animals did not use this option since they are used to people filming and not interacting with the animals during that time from other studies.

One more interesting difference that emerged between wolves and dogs is how they reacted to the motivation trials, in which the animals only had to wait for 2 s. Wolves performed overall worse than the dogs even in the motivation trials when the delay was very short. However, the performance of the wolves in the motivation trials did not decrease as the delays got longer in the associated delay trials, whereas this was the case with the dogs. These different patterns might indicate that the wolves had a lower motivation than the dogs to participate in the task from the beginning on, which might explain their worse performance in the delay trials compared to the dogs, whereas the dogs only lost their motivation with longer delays.

Alternatively, it is possible that the dogs and wolves were similarly motivated to participate in the task and their different performance in the motivation trials rather indicates that the wolves understood the task better in the sense that they learned that there are two kinds of trials: one type has very short waiting times worth waiting for and another type, where you have to continue waiting for longer times. Thus, it might be worthwhile to wait at least for 2 s, even if you do not want to wait for a long time. The imperfect performance of the wolves in the motivation trials might indicate a lack of inhibitory control, which might be reduced in the dogs due to the presence of the human (see above). However, if the wolves differentiated between the two trials, this could have also influenced their overall performance, since, with the strategy of waiting at least 2 s, they would be successful in at least half of the eight total trials, while not facing the frustration of having to wait in the longer delay trials. Moreover, by not waiting in the longer delays but in the motivation trials, they actually increased their reinforcement rate. Thus, the wolves might have employed a different, more beneficial strategy to cope with the task, explaining their worse performance in comparison to the dogs in the delay of gratification. This is a limitation in the study design that we did not anticipate and further studies should investigate, whether wolves and dogs indeed differ in how they take the reinforcement rate into account when faced with such tasks.

Finally, the fact that none of the animals—not even the wolves—differentiated between the different reward types is not very surprising in hindsight. We recently conducted a study on the food preferences of wolves and dogs and realized that neither the wolves nor dogs express a preference for meat over sausage in a two-choice task, but that they prefer both sausage and meat over dry food (Rao et al. 2018a, b).

Overall, our results offer different explanations to why dogs waited longer than wolves in this task. First, dogs are indeed better at delaying gratification than wolves at least if a human is present. This is probably best explained by direct selection for social inhibition during the process of domestication (deferential behaviour hypothesis). It would be interesting to investigate whether the dogs and the wolves would perform more similarly if the task would be conducted in an asocial manner. Second, it is also possible that the animals had a different motivation to participate in the task or third, a different understanding of the task contingencies. Future studies need to disentangle these different explanations.

## Electronic supplementary material

Below is the link to the electronic supplementary material.

Supplementary file1 (XLSX 106 kb)

Supplementary file2 (M4V 15762 kb)
